# Editorial: Genetics of Complex Traits and Diseases From Under-Represented Populations

**DOI:** 10.3389/fgene.2021.817683

**Published:** 2022-01-17

**Authors:** Segun Fatumo, Tinashe Chikowore, Karoline Kuchenbaecker

**Affiliations:** ^1^ The African Computational Genomics (TACG) Research Group, MRC/UVRI and LSHTM, Entebbe, Uganda; ^2^ The Department of Non-communicable Disease Epidemiology, London School of Hygiene and Tropical Medicine, London, United Kingdom; ^3^ Sydney Brenner Institute for Molecular Bioscience, Faculty of Health Sciences, University of the Witwatersrand, Johannesburg, South Africa; ^4^ MRC/Wits Developmental Pathways for Health Research Unit, Department of Paediatrics, Faculty of Health Sciences, University of the Witwatersrand, Johannesburg, South Africa; ^5^ Division of Psychiatry, University College of London, London, United Kingdom; ^6^ UCL Genetics Institute, University College London, London, United Kingdom

**Keywords:** genomic, underrepresentation, GWAS, genome-wide association study, genetic, complex disease

The Inclusion of ethnically diverse populations in genetic research can provide deep insights into specific pathogenic variants that differ across and within populations. Such insights may be useful to guide diagnosis and provide relevant clinical interventions (Gurdasani et al., 2019; Chikowore et al., 2021; Fatumo et al., 2021a; Fatumo et al., 2021b). Unfortunately, the vast majority of the genetic studies have been performed in European ancestry populations and few in Africa (Kuchenbaecker et al., 2019; Chikowore et al., 2021).

We accepted only high-quality research work in this research topic, including review manuscripts describing the genetics of diseases in underrepresented populations such as African, Asian, Latin American, and other indigenous populations. Topics could include Genome-wide association studies, Gene-environment interactions, Population genetics, epigenetics, pharmacogenomics, Polygenic/Genetic Risk Scores. The final topic issue has 10 published articles out of 18 submitted manuscripts, covering various genetic studies in African, Asian, Arab, and other previously under-studied populations.

Most of the studies demonstrated that better representation of ethnically and ancestrally diverse populations is crucial for our ability to comprehensively characterise the genetic architecture of complex traits and human disease. For example, Font-Porterias et al. analysed genome-wide array data and exome sequences of 89 healthy Spanish Roma individuals to investigate chronic diseases and traits. The Roma represents the largest trans-national minority ethnic group in Europe, originating from South Asia and receiving extensive gene flow from West Eurasia. The authors found frequency differences between the Roma and other non-Roma populations for disease-causing variants and variants linked to drug response. The work demonstrated the value of merging population genetics and genetic epidemiology methodology.

Other papers demonstrated how such differences in clinically relevant genetic variation can impact health inequalities, if populations are not sufficiently represented in genetic research. Chande et al. analysed the effect of genetic ancestry and ethnicity on observed disease prevalence. They predicted disease risk in Colombia populations by mainly comparing the disease prevalence and socioeconomic indicators of Colombia’s three major ethnic groups; from Mestizo, Afro-Colombian, and the Indigenous group. The study demonstrated a strong link between ethnicity, ancestry, and health outcomes. In their mini review Nagaraj and Toombs discussed “the history of pharmacogenomics and highlight the inequalities that must be addressed to ensure equal access to pharmacogenomic-based healthcare and reviewed the efforts to conduct the pharmacogenomic profiling of chronic diseases among Australian Indigenous populations including the impact of the lack of drug safety-related information on potential ADRs among individuals in these Australian Indigenous communities”.

Papers in this special issue actively addressed underrepresentation by investigating the genetics of complex traits in diverse populations. One approach used to address the imbalance is synthesising existing studies to empower more generalizable conclusions. Soremekun et al. meta-analysed summary statistics from genome-wide association studies of multiple African data, carried out a Bayesian fine-mapping, and assessed the causal relationship between White Blood Cell (WBC) subtypes and Asthma including a two-sample Mendelian Randomization (MR) analysis. This study identified five novel genes not previously reported associated with any WBC subtype. Their MR analysis showed that monocytes count and neutrophils counts are associated with an increased risk of asthma. Singh et al. conducted a systematic review to summarise studies on genetic association studies for blood pressure-related traits in populations with African ancestry. The review highlighted the limited number of available studies, especially in Africa. Nevertheless, across the five blood pressure-related traits, they found that 26 genome-wide significantly associated SNPs have been identified, including 12 associations that have not previously been described in non-African studies.

Similarly, Al-Homedi et al. leveraged several small-scale genome-wide association studies of metabolic syndrome (MetS) performed in Arab populations to investigate the genetic basis of MetS in the population. Their systematic review and meta-analysis identified 36 studies and shows that the most frequently studied genes were FTO and VDR. The study failed to find any unique genetic association between MetS and Arabian populations.

It may be unsurprising that other studies in this Special Issue focussed on cardiometabolic outcomes, given the immense public health burden of cardiovascular disease. Dlamini et al. investigated the associations between genetic variation in CYP17A1 and SERPINA6/A1 and circulating glucocorticoid concentrations in black South African adults. The authors found that a variant rs17090691 with G effect allele at *SERPINA6/A1* was associated with increased diastolic blood pressure in all adults at *p*-value of 9.47 × 10^–6^. However, no association was seen between these genetic variants and glucocorticoids or between any variants in *CYP17A1* and metabolic outcomes after adjusting for multiple testing.

In Wang et al., the authors recruited over 1,400 Han Chinese patients with coronary artery disease. They conducted linear regression analyses on the genetic variants of mitochondrial DNA (mtDNA) and lipidomic traits in two independent groups where they found three associations. They also explored the associations between genetic variants in mitochondrial and lipids traits and reported two significant associations. The study provided “insights into the lipidomic context of mtDNA variations and highlighted the importance of studying mitochondrial genetic variants related to lipid species”.

Two of the studies focused on mental health genetics in African populations. Genetic research could play an important role to decrease stigma and better understand the causes of this highly prevalent, yet poorly understood global health problem. Owalla et al. investigated the effects of stress and the genetic variation within the FK506 binding protein 5 (rs1360780) and glucocorticoid receptor (rs10482605) genes on internalizing disorders (ID) status in a CA-HIV cohort in Uganda. Using logistic regression, the authors assessed the relationships between IDs and recent stress, chronic stress, and the investigated genotypes. They reported no significant association between IDs and rs1360780 and rs10482605 polymorphisms within the FKBP5 and glucocorticoid receptor genes nor observed any gene–environment effect on vulnerability IDs in the Ugandan population, but shows that severe recent stress increases the vulnerability to IDs among CA-HIV. The findings further supported that polymorphisms at genetic loci only contributed minimal to the genetic vulnerability to IDs.

Similarly, Kalungi et al. assessed the relationships between acute stress and Internalizing mental disorders (IMDs), and moderation by chronic stress and SNPs in *SLC6A4* and *TPH2* in Ugandan populations. The authors observed a “statistically significant association between severe acute stress and IMDs. Acute stress was found to interact with *5-HTTLPR*-rs25531*S-A-S-A* haplotype to increase the risk for IMDs among Ugandan HIV^+^ children and adolescents but no evidence for a combined interaction of acute stress, chronic stress, and *5-HTTLPR*-rs25531 on IMDs”.

Overall, the studies in this Special Issue highlighted the importance of genetic research in diverse global populations. They synthesized existing data to enable conclusions for diverse populations, demonstrate population variation linked to clinically relevant genes, discover novel genetic associations, and investigate the implications for health inequalities.
